# Decreased Methane Emissions Associated with Methanogenic and Methanotrophic Communities in a Pig Manure Windrow Composting System under Calcium Superphosphate Amendment

**DOI:** 10.3390/ijerph18126244

**Published:** 2021-06-09

**Authors:** Yihe Zhang, Mengyuan Huang, Fengwei Zheng, Shumin Guo, Xiuchao Song, Shuwei Liu, Shuqing Li, Jianwen Zou

**Affiliations:** 1 Jiangsu Key Laboratory of Low Carbon Agriculture and GHGs Mitigation, College of Resources and Environmental Sciences, Nanjing Agricultural University, Nanjing 210095, China; 2020203073@stu.njau.edu.cn (Y.Z.); 2020203074@stu.njau.edu.cn (M.H.); 2019103090@njau.edu.cn (F.Z.); 2019103078@njau.edu.cn (S.G.); swliu@njau.edu.cn (S.L.); jwzou21@njau.edu.cn (J.Z.); 2 Institute of Agricultural Resources and Environment, Jiangsu Academy of Agricultural Sciences, Nanjing 210014, China; xiuchao103@163.com; 3 Jiangsu Key Lab and Engineering Center for Solid Organic Waste Utilization, Jiangsu Collaborative Innovation Center for Solid Organic Waste Resource Utilization, Nanjing Agricultural University, Nanjing 210095, China

**Keywords:** methane (CH_4_), calcium superphosphate (CaSSP), manure composting, methanogens, methanotrophs, *Methanosarcina*

## Abstract

With the rapid growth of livestock breeding, manure composting has evolved to be an important source of atmospheric methane (CH_4_) which accelerates global warming. Calcium superphosphate (CaSSP), as a commonly used fertilizer, was proposed to be effective in reducing CH_4_ emissions from manure composting, but the intrinsic biological mechanism remains unknown. Methanogens and methanotrophs both play a key role in mediating CH_4_ fluxes, therefore we hypothesized that the CaSSP-mediated reduction in CH_4_ emissions was attributed to the shift of methanogens and methanotrophs, which was regulated by physicochemical parameter changes. To test this hypothesis, a 60-day pig manure windrow composting experiment was conducted to investigate the response of CH_4_ emissions to CaSSP amendment, with a close linkage to methanogenic and methanotrophic communities. Results showed that CaSSP amendment significantly reduced CH_4_ emissions by 49.5% compared with the control over the whole composting period. The decreased *mcrA* gene (encodes the α-subunit of methyl-coenzyme M reductase) abundance in response to CaSSP amendment suggested that the CH_4_ emissions were reduced primarily due to the suppressed microbial CH_4_ production. Illumina MiSeq sequencing analysis showed that the overall distribution pattern of methanogenic and methanotrophic communities were significantly affected by CaSSP amendment. Particularly, the relative abundance of *Methanosarcina* that is known to be a dominant group for CH_4_ production, significantly decreased by up to 25.3% accompanied with CaSSP addition. Only Type I methanotrophs was detected in our study and *Methylocaldum* was the dominant methanotrophs in this composting system; in detail, CaSSP amendment increased the relative abundance of OTUs belong to *Methylocaldum* and *Methylobacter*. Moreover, the increased SO_4_^2−^ concentration and decreased pH acted as two key factors influencing the methanogenic and methanotrophic composition, with the former has a negative effect on methanogenesis growth and can later promote CH_4_ oxidation at a low level. This study deepens our understanding of the interaction between abiotic factors, function microbiota and greenhouse gas (GHG) emissions, as well as provides implication for practically reducing composting GHG emissions.

## 1. Introduction

Methane (CH_4_) is the second largest contributor to the radiative forcing of the atmosphere, and its global warming potential is 25 times that of carbon dioxide (CO_2_) on a mass basis in a 100-year time frame [1]. The CH_4_ emissions from livestock production accounted for 12–41% of the total agricultural CH_4_ emissions [2,3]. In China, CH_4_ emissions from manure management were estimated to be 3.2 Tg in 2014, accounting for 14.2% of agricultural total of CH_4_ emissions [4]. According to the “Technical specification for sanitation treatment of livestock and poultry manure” issued in 2018 [5], composting can be the key technology for the sanitation and recycling of animal manure and anaerobic digestate due to its cost-effective advantages. Additionally, compost also provides a vital link in ecological cycle agriculture model combining planting and breeding, however, its contribution to CH_4_ emissions is of increasing concerns [6,7].

The CH_4_ emissions from manure composting systems is a microbe-dominated process that depends on the balance between CH_4_ production and oxidation [8,9]. Generally, pathways of methanogens for generating CH_4_ included: (A) methanogenesis from H_2_/CO_2_ or formate; (B) methanogenesis from methanol; and (C) methanogenesis from acetate [10]. On the other hand, methanotrophs are responsible for CH_4_ oxidation, which can utilize CH_4_ as carbon source and energy, and all known species of methanotrophs were divided into two types, Type I and Type II, belonging to phylum proteobacteria in the relative classes Gammaproteobacteria and Alphaproteobacteria [11]. To analyze and quantify methanogenic and methanotrophic communities, the highly conserved *mcrA* and *pmoA* genes encoding the α-subunits of methyl-coenzyme M reductase and particulate membrane bound methane monooxygenase have been widely used [9,12,13]. Collectively, the combined performance of both methanogenic and methanotrophic microorganisms together contributes to the net yield of CH_4_ emissions. Numerous studies have focused on the methanogens and methanotrophs in paddy soils, water environments and rumen systems [14,15,16]. Unfortunately, few experiments have concentrated on the diversity and composition of methanogens and methanotrophs in manure composting systems, even though manure compost is an important source of CH_4_ [17].

Calcium superphosphate (CaSSP), as a commonly used fertilizer, was proposed to be effective in reducing CH_4_ emissions and improving the quality of the final product during the manure composting process [18]. In addition, the amendment of CaSSP may affect the physiochemical properties (e.g., pH, SO_4_^2−^ and temperature) which way induce changes in microbial community composition and their activities [19]. Peng et al. [20] has shown that in a chicken manure compost, lower pH caused by calcium superphosphate addition can significantly affect the main bacterial families. Furthermore, the increase in SO_4_^2−^ concentration can also inhibit methanogens growth due to the competition for organic carbon and energy source [21]. However, in manure composting, more research is needed to conduct a study about the intrinsic biological mechanism in mitigating CH_4_ emissions, that is what are the effects of CaSSP addition on the population and community of methanogens and methanotrophs that are sensitive to the changes in abiotic factors, and particularly, it remains unclear how the functional microbial communities interact with those abiotic factors in reducing CH_4_ emissions from manure composting [18,22]. Therefore, an insight into the behavior of methanogenic and methanotrophic communities following CaSSP amendment in manure composting is expected to better understand the potential mechanisms for driving GHG mitigation.

An in situ measurement of CH_4_ fluxes from a windrow manure composting system was conducted to investigate the effects of CaSSP on CH_4_ production and oxidation. The q-PCR and Illumina MiSeq sequence methods were used to measure the abundance and composition of methanogenic and methanotrophic communities during the composting process. The main objectives of this study were first to examine the abundance and communities of methanogenic and methanotrophic in response to CaSSP amendment in manure composting system; we also attempted to explore the linkage between the key abiotic factors and CH_4_-related functional microbes. We hypothesized that the CH_4_ emissions reduced following CaSSP amendment in manure composting systems could be closely associated with the combined performance of methanogenic and methanotrophic communities (i.e., abundance, composition, and activity), which are mediated by the altered physicochemical characteristics.

## 2. Materials and Methods

### 2.1. Windrow Composting Construction

The windrow composting experiment was conducted in a fertilizer company, located at Nanjing, Jiangsu province, China. The detailed composting design was referred to by Jin et al. [23]. Two treatments were designed including the control without calcium superphosphate (CaSSP) addition, and the CaSSP treatment receiving the amendment at a weight of 5% (*w*/*w*, DW) of the raw composting materials. Three replicate windrow composting piles were constructed for each treatment using a mixture of pig manure solids and straw with a mixing ratio of 6:1 on a dry weight basis. The size of the windrow in this study was about 12 m × 1.5 m × 1 m (length × width × height). Gas sampling was carried out for each pile separately, and in addition to that, composting materials samples were collected near the positions of gas sampling.

### 2.2. Measurement of CH_4_ Fluxes

Static chamber-gas chromatograph (GC) method was carried out to measure the CH_4_ fluxes during the composting progress [24,25]. Gas samples were collected once or twice each week. The PVC chamber collar bases (0.3 m length × 0.3 m width × 0.25 cm height) were pre-inserted 0.25 m into the pile 10–12 h before each gas sampling to minimize the disturbance [18]. The top edge of the collar had a groove (5 cm in depth) for filling with water to ensure the sealing property of the device. The opaque chamber was designed as a size of 0.3 m long × 0.3 m wide × 0.5 m tall. While gas sampling, the chamber was placed on the top of each air grid, and the edges of the chambers coincided with the grooves in the collar. Gas samples were collected at 0, 5, 10, 20 and 30 min after chamber closure between 8:00 and 10:00 am on each sampling day, respectively [25,26,27]. After gas sampling, the samples were then immediately transported to the laboratory for analysis by gas chromatograph (GC).

The mixing ratio of CH_4_ was analyzed by a modified gas chromatograph (Agilent 7890, Santa Clara, CA, USA) which was equipped with a flame ionization detector (FID) [28,29]. The oven was operated at 55 °C while the FID was at 200 °C. The flow rate of carrier gas (N_2_) was 30 mL min^−1^. The average flux which was taken from three parallel sections within each windrow represented the flux measurement of the sampled grid on each sampling day. For each treatment, average fluxes, and standard deviations of CH_4_ fluxes were calculated from three replicate windrows. CH_4_ emissions during composting were sequentially accumulated from the fluxes between two adjacent measurement intervals [28,29,30].

### 2.3. Physicochemical Parameters Determination

At each time for gas flux sampling, 300 g of composting samples were also collected and divided into three parts. The first part was air-dried and ground to pass through a 1 mm sieve and 2 mm sieve for pH, total carbon (TC) and total nitrogen (TN) analyses, respectively. The second part was used to analyze soil-based physicochemical properties. The remaining part was used at −80 °C for molecular analysis. Soil pH was determined with a soil-to-water ratio of 1:2.5 by pH electrode (PHS-3C mv/pH detector, Shanghai, China). The moisture content of each fresh sample was determined by constant weight loss upon drying at 105 °C for 24 h. Soil nitrate (NO_3_^−^-N) concentrations were measured following two-wavelength ultraviolet spectrometry at 220 and 275 nm, and ammonium (NH_4_^+^-N) concentrations were measured using the indophenol blue method (HITACHI, U-2900, Tokyo, Japan). The C/N ratio was calculated based on the total carbon (TC) and total nitrogen (TN) concentrations which were determined by an auto elemental analyzer (Vario EL III, Elementar, Hanau, Germany). Dissolved organic carbon (DOC) was determined by ultraviolet-enhanced persulfate digestion and infrared detection (Phoenix 8000, Teledyne Tekmar, Cincinnati, USA) The data of physicochemical parameters are listed in Table S1 and referenced Jin et al. [23].

### 2.4. DNA Extraction and Real-Time Quantitative PCR (q-PCR) Assays of the Functional Genes

During the composting time, manure samples (days 1, 10, 17, 24, 31 and 38) were selected for DNA extraction at which time the gas flux was sampled. DNA samples were extracted from manure (0.5 g) with the MoBio PowerSoil^TM^ DNA Isolation Kits (Mo Bio Laboratories, Carlsbad, CA, USA). The concentration of DNA samples was determined by a Nanodrop (Thermo Scientific, Waltham, MA, USA). After that, the DNA samples were used for quantitative polymerase chain reaction (qPCR) and Illumina Miseq sequencing analysis.

Real-time quantification of *mcrA* and *pmoA* genes was conducted in a StepOne^TM^ real-time PCR system (Applied Biosystems, Foster City, USA). The copy number of the *mcrA* and *pmoA* genes used the primer pair mals/*mcrA*-rev [31] and A189F/Mb66R [32], respectively. The q-PCR amplifications were performed in a total volume of 20 μL using a SYBR^@^ Premix Ex Taq^TM^ (Takara, Dalian, China), with a reaction mixture which consisted of 10 μL SYBR@ Premix Ex Taq™ (Takara, Dalian, China), 0.4 μL each primer (10 μmol L^−1^), 0.4 μL ROX reference dye (50×), 2 μL template DNA and 6.8 μL sterile water. The amplified fragments for each gene were cloned in pMD 18-T vector and sequenced. The standard curves of both genes were prepared by using triplicate 10-fold dilutions of linear plasmid DNA. Amplification was performed in triplicate under the following cycling conditions: 30 s at 95 °C, sequenced by 40 cycles of 95 °C, 55 °C and 72 °C for 5 s, 30 s, and 15 s, respectively, and eventually a dissociation stage by a dissociation stage at 95 °C for 15 s, 55 °C for 30 s, and 95 °C for 15 s [17,33].

### 2.5. Illumina MiSeq Sequencing of mcrA and pmoA Genes

The community structure of methanogens and methanotrophs were assessed by Illumina MiSeq sequencing of *mcrA* and *pmoA* genes, respectively. The mals/*mcrA*-rev [31] and A189F/Mb66R [34] primers were set up for the amplification of *mcrA* and *pmoA* genes, respectively. The Axyprep DNA Gel Extraction Kit (Axygen Biosciences, Union City, CA, USA) was used to purify the targeted bonds (approximately 410 bp for *mcrA* and 478 bp for *pmoA*), and QuantiFluor^TM^-ST (Promega, Madison, WI, USA) was employed to quantify the targeted bonds. For each sample, the PCR was repeated in triplicate, after that the three PCR products were mixed together. The PCR products of each DNA sample were evaluated by 2% agarose gel electrophoresis. The equimolar purified amplicons were pooled, and pair-end sequenced (2 × 250) on the Illumina MiSeq platform according to the standard protocols at Shanghai BIOZERON Biotechnology Co., Ltd. (Shanghai, China). The Illumina Miseq sequencing data are available in the NCBI Sequence Read Archive (SRA) database under accession number PRJNA661995.

### 2.6. Analysis of Illumina MiSeq Sequencing Data

According to Caporaso et al. [35], three criteria were followed for demultiplexing and quantity-filtering the raw *mcrA* and *pmoA* sequences by Quantitative Insights Into Microbial Ecology (QIIME) (Version 1.17) after sequencing was completed: (1) sequences with reads shorter than 200 bp and a quantity score below 25 were discarded from further analysis; (2) sequences were clustered into operational taxonomy units (OTUs) at 97% identity threshold; (3) chimeric sequences were identified and removed by using UCHIME [36]. The *mcrA* sequences were binned into species-level OTUs at 84% sequence identity which corresponded with 97% similarities based on 16S rRNA gene by using UCLUST [37] while *pmoA* sequences which shared 87% similarity (corresponding to 97% similarities based on 16S rRNA gene) were also binned into species-level OTUs with UCLUST [38]. The phylogenetic affiliation analysis of each *mcrA* and *pmoA* gene sequence was introduced by RDP Classifier against the silva 104 database (http://www.arb-silva.de/download/archive/qiime/ (accessed on May 10 2019)) with a confidence threshold of 70%.

To calculate the alpha-diversity of a methanogen community, the abundance-based coverage estimator (ACE), Chao 1 estimator, Shannon diversity and Good’s coverage were calculated. Principal co-ordinates analysis (PCoA) was carried out to express the relationship between the structure of methanogenic and methanotrophic communities and CH_4_ fluxes.

### 2.7. Statistical Analysis

One-way analysis of variance (ANOVA) was performed to examine the effects of CaSSP on soil chemical properties over the composting time. A pairwise correlation was conducted for each pair of variables, including CH_4_ fluxes, the abundance of functional genes, physicochemical parameters, and the relative abundance of genera. Statistical significance was determined at the 0.05 probability level. A linear model with ordinary least squares (OLS) was used to fit the correlations of CH_4_ fluxes with methanogenic and methanotrophic abundances. A *t*-test was used to examine the statistical significance of parameter estimates in the simulated OLS model. All target variables are expressed as means of replicates. A permutational multivariate analysis of variance (PERMNOVA) was conducted using the Adonis function in R vegan package with 999 permutations to test the significance of factors which accounted for the divergence in communities of methanogen and methanotroph (standard Mantel test). Bray–Curtis-based principal co-ordinates analysis (PCoA) was employed to analyze the microbial community compositions and the treatments. Statistical analyses were carried out by SPSS 20.0 (IBM, Armonk, NY, USA) and R.

## 3. Results

### 3.1. CH_4_ Fluxes

**Figure 1:**
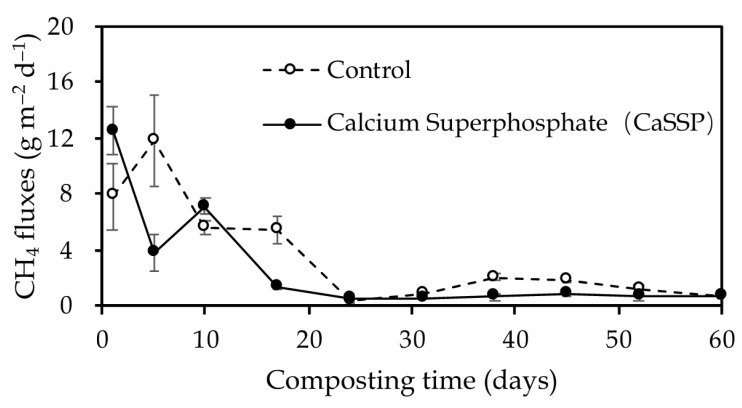
The dynamics of CH_4_ fluxes during the composting progress. Error bars show a standard error of the mean of triplicate compost windrows.

During the 60-day composting, the similar trend of CH_4_ fluxes between control and CaSSP treatment was observed explicitly (Figure 1). The CH_4_ fluxes increased steadily to the largest fluxes on approximately 4–7 days after composting. After two weeks of composting, the CH_4_ fluxes showed a steady decrease until the end of the composting system. Total CH_4_ emissions mainly occurred during the early heating stage (1–10 days) of the compost, which accounted for 49.5–56.6% of the whole composting period. Compared with the control pile, the application of CaSSP significantly decreased the cumulative CH_4_ emissions by 33.80% (*p* < 0.05 by *t*-test) (128.27 g·m^−2^ vs. 193.75 g·m^−2^).

### 3.2. Methanogenic and Methanotrophic Abundances

**Figure 2:**
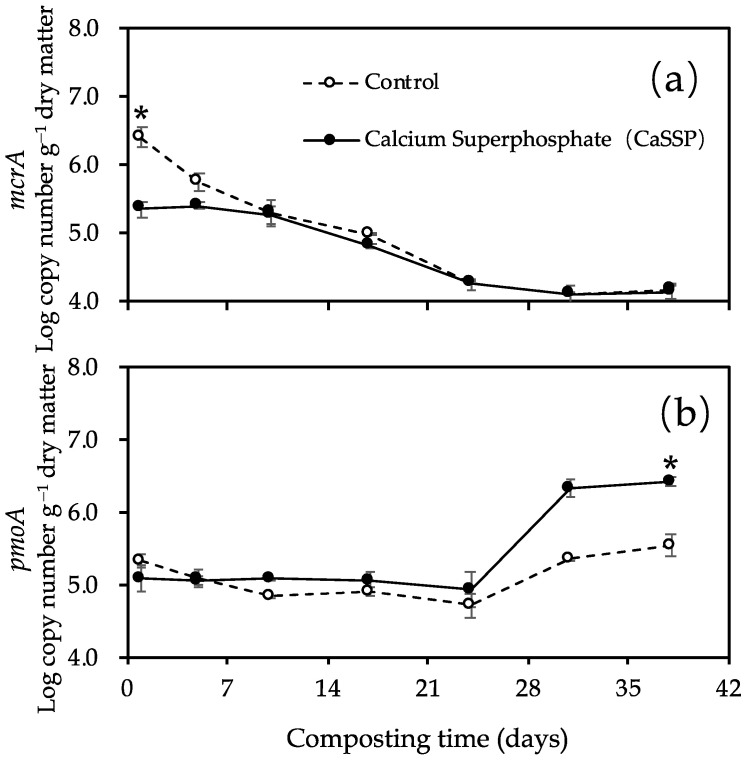
The *mcrA* (encodes the α-subunit of methyl-coenzyme M reductase) (**a**) and *pmoA* (encodes the α-subunit of particular membrane bound methane monooxygenase) (**b**) genes copy numbers as affected by CaSSP amendment during windrow manure composting. Control, manure composting; CaSSP, manure composting in combination with CaSSP. Vertical bars indicate standard errors of three replicates. * indicates statistically significant at *p* < 0.05 by *t*-test.

**Figure 3:**
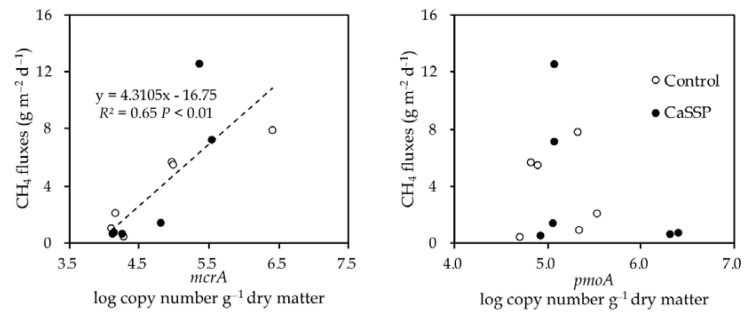
Dependency of CH_4_ fluxes on the abundance of *mcrA* (**a**) and *pmoA* (**b**) genes across different treatments. Treatments were defined as in Figure 1. SSP: superphosphate.

The abundances of *mcrA* and *pmoA* genes showed a trade-off trend over the composting system (Figure 2a,b). The largest *mcrA* abundance was measured at Day 1 of composting, then gradually decreased and was maintained at low levels until the end of composting progress. On the contrary, the copy number of *pmoA* gene increased at the cooling and maturing phase of composting progress (after Day 24). The significant difference in *mcrA* copy number between two treatments was shown at the beginning of the compost (Day 1) while the *pmoA* copy number differed at the end of the composting (Day 38) (*p* < 0.05 by *t*-test). Furthermore, CaSSP amendment decreased the abundance of *mcrA* by 113.6% on Day 1 while increasing the abundance of *pmoA* by approximately 150% on Day 38. In the whole composting process, CH_4_ fluxes showed a positive correlation (r^2^ = 0.65, *p* < 0.01 by *t*-test) with *mcrA* abundance but showed no significant association with *pmoA* abundance across the two treatments. (Figure 3a,b). To be more specific, the difference in the abundance of *mcrA* appeared at the beginning of the compost in which time the dominant CH_4_ emissions occurred. Importantly, CaSSP addition caused the significant alternation of SO_4_^2−^ concentration and pH at this stage (*p* < 0.05 by *t*-test) (Table S1), therefore we can speculate that these two can be key factors inhibiting *mcrA* abundance. However, at the end of the compost, the *mcrA* abundance in two treatments were both kept low, thus we cannot detect the correlation between *mcrA* abundance and SO_4_^2−^ concentration or pH. Pairwise correlations indicated that *mcrA* abundance was significantly related to TC, C/N and DOC (Table S2), while all of the three parameters were rarely different between the two treatments (Table S1), suggesting they may not be the dominant factors involved in the CaSSP-repressed *mcrA* abundance (but maybe involved in the composting stage-mediated *mcrA* abundance alternation).

### 3.3. Response of Methanogenic and Methanotrophic Community to CaSSP Addition

**Figure 4:**
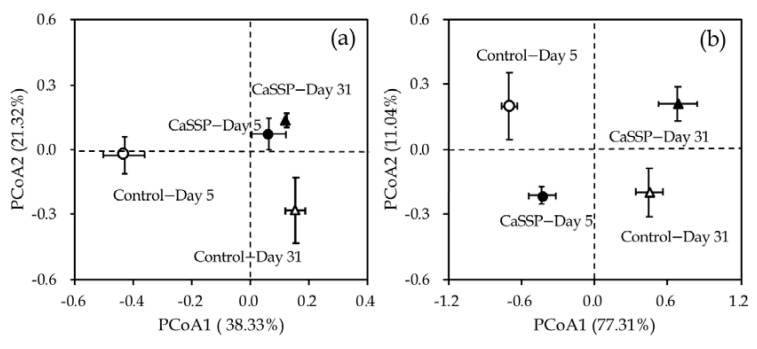
Methanogenic (**a**) and methanotrophic (**b**) community structures (based on relative OTU abundance) assessed by principal coordinate analysis (PCoA) based on the Bray–Curtis distance for different treatments. Treatments were defined as in Figure 1.

**Figure 5:**
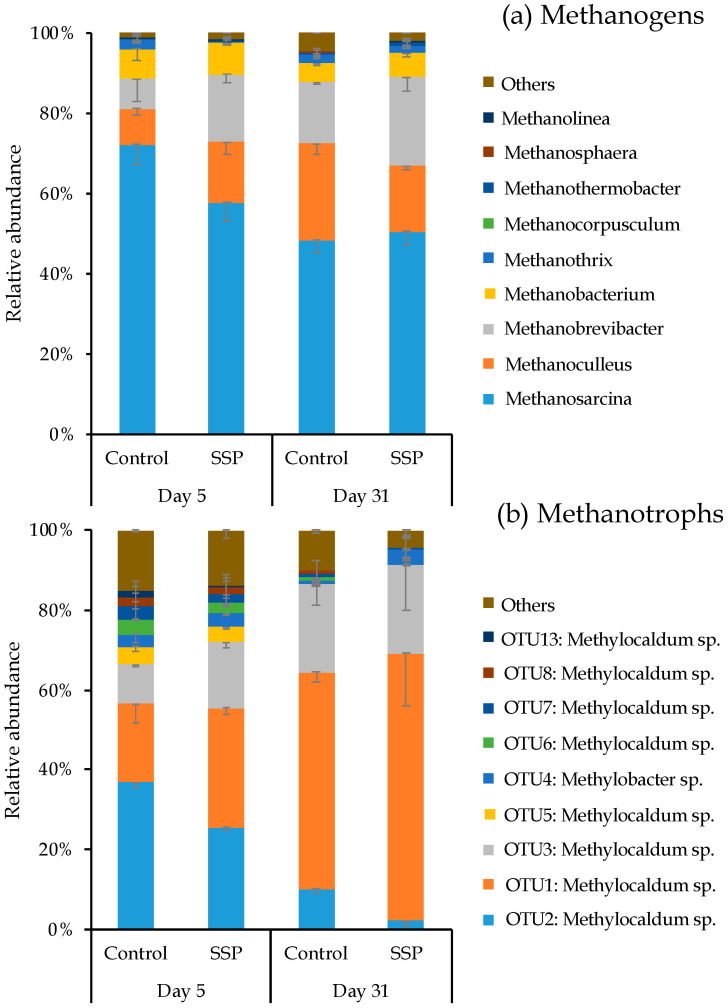
Relative abundance of methanogens (**a**) and methanotrophs (**b**) under different fertilization treatments. Relative abundance of methanogens and methanotrophs for the taxonomic levels of genus and OTU, respectively. Values are means ± SE (*n* = 3). Treatments were defined as in Figure 1.

Methanogenic and methanotrophic community structures were determined by Illumina MiSeq sequencing for *mcrA* and *pmoA* genes, respectively. CaSSP amendment did not significantly alter the α-diversity of methanogenic community (Table S3). Principal co-ordinates analysis (PCoA) recovered the differences of methanogenic and methanotrophic communities among two composting treatments (Figure 4a). The first principal component (38.33% of the contribution rate) differentiates the methanogenic communities of the control and CaSSP treatment in the bio-oxidative phase of the composting progress (Day 5), whereas non-significant variations in methanogenic community were observed on Day 31. The top nine abundant methanogenic genus in composting occupied almost 98% of all methanogenic groups on average (Figure 5a). *Methanosarcina* (48.3~72.3%), *Methanoculleus* (8.8~24%) and *Methanobrevibacter* (7.4~22.2%) were the three dominant genera in methanogenic community in the two composting phases (Day 5 and Day 31) of composting progress. Compared with Day 5, the relative abundance of *Methanosarcina* genus decreased by 35.3% on average in two treatments on Day 31, while *Methanoculleus* and *Methanobrevibacter* revealed higher relative abundances on Day 31 than Day 5. Importantly, the bio-oxidative phase (Day 5) that served as the dominant stage for CH_4_ emissions, and the CaSSP amendment distinctly decreased the relative abundance of *Methanosarcina* up to 25.3% as compared with the control. The relative abundances of these three dominant methanogen genera were similar between the two treatments on Day 31.

CaSSP amendment significantly decreased the α-diversity of methanotrophs at the cooling and maturing phase of the composting progress, as supported by ACE, Chao1 and Shannon indexes (*p* < 0.05 by *t*-test) (Table S3). For the methanotrophic composition, PCoA results revealed that about 90% of the total variability can be explained by the first two axes (Figure 4b). The results of PCoA showed that different periods of compost can affect methanotrophs. According to the phylogenetic classification, the top-9 abundant methanotrophic OTUs in this composting system all belonged to *Methylocaldum* and *Methylobacter* genus and accounted for approximately 90% of the methanotrophic community (Figure 5b). The relative abundances of these two genera were higher on the cooling and mature phase (Day 31) as compared with the bio-oxidative phage (Day 5). *Methylocaldum* (81.65~91.85%) was identified to be the most predominant group of methanotrophic community in this pig composting system (Figure 5b). Compared with the control, the CaSSP amendment did not alter the compositions of methanotrophic community at the genus level in the composting progress. However, in line with the results of standard Mantel test and PCoA analysis based on the OTU of methanotrophs, we speculate that different OTUs such as OTU1 and OTU2 belonging to *Methylocaldum* may have various degrees of influence on CH_4_ oxidation.

Furthermore, we performed a Mantel test to prove that both methanogenic and methanotrophic communities were significantly correlated with CH_4_ fluxes (999 unrestricted random permutations; *p* < 0.05; Table S4), revealing that the contribution of microbial composition shifting in gaseous mitigation. Within different physicochemical parameters during the composting (Table S1), TN, C/N, SO_4_^2−^ and DOC revealed a significant relationship with the methanogen community, while NH_4_^+^-N, pH, TN, TC and C/N were observed to correlate with methanotrophic community (Table S4). Importantly, only pH, NH_4_^+^-N and SO_4_^2−^ were shown to be significantly different across the two treatments (Table S1), implicating that they are likely to be involved in the CaSSP-affected microbial community shifting and CH_4_ mitigation.

## 4. Discussion

Windrow composting is one of the popular large-scale composting strategies, owing to its convenient and relatively lower cost [39]. In this study, the CaSSP-mediated significant reduction in CH_4_ fluxes during the composting progress was similar to previous reports [18,40,41], and both the emission peaks and substantial mitigation were observed at the bio-oxidative phase (Figure 1). The CH_4_ mitigation of CaSSP amendment could be attributed to the community shifts of methanogens and methanotrophs, which played important roles in CH_4_ emissions from composting [42].

### 4.1. Methane Production by Methanogenic Community

The maximal fluxes of CH_4_ from both Control and CaSSP treatments were observed in the first week (Figure 1), and the highest copy numbers of *mcrA* were also detected during the bio-oxidative phase (Figure 2a). In this phase, the decreased oxygen concentrations and increased temperatures of piles may have led to the priming effects on methanogenic activities responsible for CH_4_ production [43]. Importantly, the abundance of *mcrA* gene in CaSSP treatment were significantly lower than that in the control, which could be the main induction factor connected with lower CH_4_ emissions (*p* < 0.05 by *t*-test).

In addition to the population size of methanogens, their community structure also played a decisive role in CH_4_ production [44]. Previous research has revealed several distinct orders of methanogenic archaea associated with CH_4_ production, including *Methanobacteriales*, *Methanosarcinales*, *Methanopyrales*, *Methanocellales*, *Methanomicrobiales*, *Methanococcales* and *Methanoplasmatales* [45,46]. The predominant methanogenic orders found in our compost system were *Methanosarcinales* (50.5~74.5%), *Methanobacteriales* (15.1~29.0%) and *Methanomicrobiales* (9.2~24.4%), which were also detected in other manure systems and rice paddies amended with organic fertilizers [20,44,47]. Specifically, the genus *Methanosarcina* occupied more than 50% in the methanogenic community, suggesting that the acetoclastic methanogenic pathway may play key roles in methane production during the composting system.

**Figure 6:**
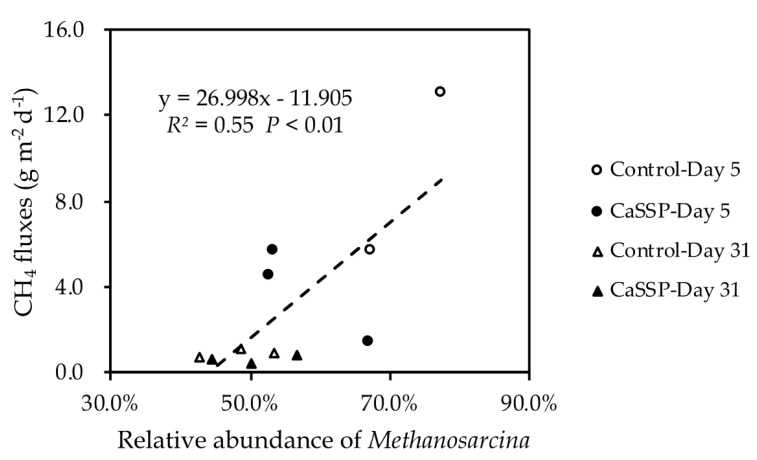
Dependence of CH_4_ fluxes on the relative abundance of *Methanosarcina* in the compost for different treatments. Treatments were defined as in Figure 1.

Compared to control piles, CH_4_ emissions in the CaSSP treatment was clearly reduced by 33.8% and this reduction could be linked with the two dominant methanogenic genera, *Methanosarcina* and *Methanobrevibacter* (Figure 5a). According to Miller et al. and Mountfort et al. [48,49], *Methanosarcina* has a higher methane production potential than *Methanobrevibacter*. A previous study has revealed that population differentiation between *Methanosarcina* and *Methanotrevibacter* can lead to the decrease in CH_4_ emissions in cattle manure composting [44], and importantly, a higher abundance of *Methanosarcina* was related to more CH_4_ emissions. Coincidentally, in our pig manure compost system, the decrease in *Methanosarcina* and increase in *Methanobrevibacter* in response to CaSSP application should announce a lower CH_4_ production activity as compared with the control, which can also be verified by the significant positive correlation between CH_4_ fluxes and *Methanosarcina* abundances (*p* < 0.05 by *t*-test) (Figure 6). Generally, it can be inferred that the composition of methanogenic communities exerts an important influence on CH_4_ production and in this pig manure composting system, *Methanosarcina*, may be the key methanogenic group for CH_4_ fluxes from composting piles.

### 4.2. Methane Oxidation by Methanotrophic Community

CH_4_ emissions of composting progresses were reduced to a low level at the cooling and mature phase in both Control and CaSSP treatments, which may be related to the increased methanotrophic populations (Figure 2b). Consistently with our results, Chen et al. [44] also found that the abundance of *pmoA* showed a stably increased trend during the whole composting time. To our knowledge, CH_4_ emissions can be mitigated by both increasing methanotrophs and decreasing methanogens [13]. However, in this study, the copy numbers of methanotrophs did not show a clear relationship with CH_4_ fluxes (Figure 3b).

As a crucial group of methylotrophic bacteria, methanotrophs can use methane as their only carbon and energy source [50]. Previous studies reported that two major groups of methanotrophs, Type I and Type II, play an important role in methane oxidation [11]. Our results show that two genera, *Methylocaldum* and *Methylobacter*, in the family of *Methylococcaceae*, were the only two genera of Type I methanotrophs that existed in Control and CaSSP treatments, while Type II methanotrophs were not detected in the composting progress. This observation was in accordance with Chen et al. [44], but in contrast to the study performed under low CH_4_ and high O_2_ conditions [51]. In the present study, no obvious differences were obtained for the methanotrophic communities at the genus level between control and CaSSP treatment (*p* > 0.05 by *t*-test).

### 4.3. Physicochemical Parameters Involved in the CaSSP Affected CH_4_ Emissions during Composting

Based on the standard Mantel test, CH_4_ fluxes showed significant correlation with the community composition of methanogenic and methanotrophs in this composting system (Table S4). It can be inferred that CH_4_ fluxes in composting progress are mainly regulated by the methanogenic community [52]. The shifts of a methanogenic community with CaSSP amendment can be attributed to the accumulation of SO_4_^2−^ (*p* < 0.05 by *t*-test) (Tables S1 and S4). Linquist et al. [53] indicated that SO_4_^2−^ has a toxic effect on methanogens because of the competitive relationship between sulfate reducing bacteria and methanogens for organic carbon and other energy sources [54]. Reductions in SO_4_^2^^−^ can reduce substrate concentrations to a level inadaptable for the growth of methanogenesis, and for example, in this compost system, with the enhancement of SO_4_^2−^ concentration, the abundance of *mcrA* was decreased in CaSSP treatment at the beginning of the compost. Furthermore, we can also infer that the decrease in the relative *Methanosarcina*, to some extent, was attributed to the enhancement of SO_4_^2−^ concentration in CaSSP treatment (Figure 5a and Table S1). Therefore, the accumulated SO_4_^2−^ concentration brought by CaSSP amendment can be a primary factor that inhibited the growth of key methanogenesis genus and decreased CH_4_ emissions.

**Figure 7:**
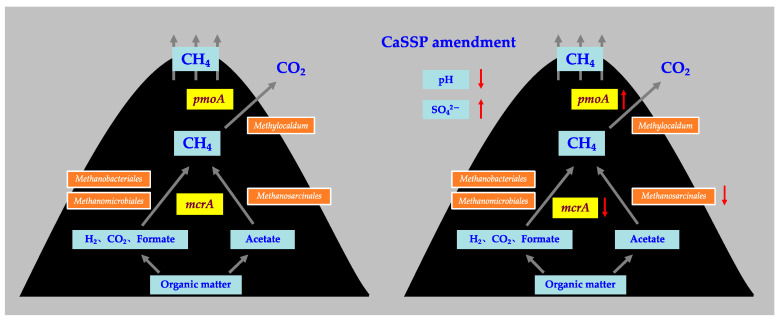
A conceptual diagram illustrating the effects of adding superphosphate on manure properties as well as methane fluxes.

In addition, compared with the control pile, the pH values of CaSSP treatment were moderately lower due to the low pH values of CaSSP (pH = 4.32) [40], which was detected to be correlated with the methanotrophic community structure and can promote CH_4_ oxidation (Table S4). Furthermore, in this study, *pmoA* abundance showed a significant and negative relationship with pH value (*p* < 0.05 by *t*-test) (Table S2). On the other hand, the increased NH_4_^+^ concentration under lower pH also contributed to CH_4_ oxidation [18,22,55]. We developed a conceptual diagram that explains how adding CaSSP affected CH_4_ fluxes: in response to CaSSP application, the alternation of physicochemical parameters such as pH and SO_4_^2−^ concentration, play the regulated role in affecting *mcrA* and *pmoA* abundance and reshaping methanogenic and methanotrophic communities, especially the key group of methanogenic community (Figure 7).

## 5. Conclusions

Calcium superphosphate (CaSSP) amendment to the compost was confirmed as an innovative management method for CH_4_ mitigation. The reduction in CH_4_ emissions with the addition of CaSSP in compost could be attributed to the increased SO_4_^2−^ concentration which can finally reduce the population of methanogens (*mcrA* abundance) and affect methanogens (e.g., *Methanosarcina*) growth. A decrease in pH value can regulate *pmoA* abundance and the succession of the methanotrophic community, although there were no significant changes in methanotrophic community composition (on the level of genus). Therefore, we suggest CaSSP can change the physiochemical properties (pH and SO_4_^2−^ had the main effect) in manure compost which affected the population and composition of methanogenic and methanotrophic community.

In this study, pH and SO_4_^2−^ played an important role in regulating the population and composition of methanogenic and methanotrophic communities after adding CaSSP in manure compost. However, there were also other driving factors (e.g., phosphorus and calcium) mitigating CH_4_ emissions that we did not detect. When conducting subsequent studies, it is necessary to consider various factors and evaluate the influences of CaSSP addition on CH_4_ production and oxidation processes comprehensively. Moreover, we observed that the methanogens and methanotrophs had a different response to CaSSP amendment on different classification levels, so laboratory experiments such as culture can be conducted for further study in the future.

## Data Availability

The sequences were submitted to the National Center for Biotechnology Information (PRJNA661995). Other data generated or analyzed during this study were included in this manuscript and Supplementary Materials.

## Supplementary Materials

The following are available online at https://www.mdpi.com/article/10.3390/ijerph18126244/s1, Table S1 Manure properties (mean ± SE, *n* = 3) from two treatments at different dates of the compost. Control, manure fertilizer, CaSSP, manure fertilizer in combination with superphosphate., Table S2 The diversity of methanogenic and methanotroph communities as affected by different fertilization during the compost. Different letters among treatments indicate significant difference at *p* < 0.05 possibility level., Table S3 Methanogenic and methanotrophic community structures for tests of manure properties using the standard Mantel test.
