# Supplementary material for: Decreased Methane Emissions Associated with Methanogenic and Methanotrophic Communities in a Pig Manure Windrow Composting System under Calcium Superphosphate Amendment
Source: Int J Environ Res Public Health. 2021 Jun 9;18(12):6244. doi: 10.3390/ijerph18126244 (PMC8296093; doi:10.3390/ijerph18126244)
Supplement: Supplementary file 1 [file ijerph-18-06244-s001.zip › ijerph-1194700-supplementary.pdf]

Decreased methane emissions associated with methanogenic and methanotrophic communities in a pig manure windrow composting system under calcium superphosphate amendment

Yihe Zhang[1], Mengyuan Huang[1], Fengwei Zheng[1], Shuming Guo[1], Xiuchao Song[3], Shuwei Liu[1,2], Shuqing Li[1,2*] and Jianwen Zou[1,2]

Author affiliation:

[1]Jiangsu Key Laboratory of Low Carbon Agriculture and GHGs Mitigation, College of Resources and Environmental Sciences, Nanjing Agricultural University, Nanjing 210095, China

[2]Jiangsu Key Lab and Engineering Center for Solid Organic Waste Utilization, Jiangsu Collaborative Innovation Center for Solid Organic Waste Resource Utilization, Nanjing Agricultural University, Nanjing 210095, China

[3]Institute of Agricultural Resources and Environment, Jiangsu Academy of Agricultural Sciences, Nanjing 210014, China

Corresponding author (Shuqing Li)

*Phone: +86 25 8439 6286; fax: +86 25 8439 5210; e-mail: shuqingli@njau.edu.cn.

To be submitted to International Journal of *Environmental Research and Public Health*

Table S1

Manure properties (mean ± SE, n=3) from two treatments at different dates of the compost. Control, manure fertilizer, CaSSP, manure fertilizer in combination with superphosphate.

Days	Treatments	pH	NH_4^+-N (g kg^{-1} DM	NO_3^--N (g kg^{-1} DM)	DOC (mg kg^{-1} DM)	TN (%)	TC (%)	C/N	SO_4^{2-} (mg g^{-1} DM)
1	Control	7.23 ± 0.07	2.33 ± 0.09	0.33 ± 0.01	40.78 ± 1.05	1.57±0.06	33.48±0.55	21.41 ± 1.15	3.66 ± 0.07
	SSP	7.30 ± 0.08	2.74 ± 0.08*	0.29 ± 0.01	43.83 ± 1.19	1.57±0.04	21.11±0.62*	20.51 ± 0.33	17.68 ± 0.03*
5	Control	7.34 ± 0.01	2.62 ± 0.06	0.30 ± 0.03	38.01 ± 0.52	1.47±0.04	31.96±0.04	21.73 ± 0.65	3.51 ± 0.02
	SSP	7.20 ± 0.04*	2.80 ± 0.07	0.25 ± 0.02	36.24 ± 0.74	1.60±0.05	31.86±0.20	20.01 ± 0.81	17.20 ± 0.09*
10	Control	7.50 ± 0.05	3.00 ± 0.07	0.26 ± 0.02	37.51 ± 0.71	1.36±0.05	29.98±0.14	22.05 ± 0.85	3.52 ± 0.09
	SSP	7.08 ± 0.18*	2.85 ± 0.08	0.22 ± 0.02	36.06 ± 1.21	0.65±0.05*	31.48±0.70	19.17 ± 0.94	16.31 ± 0.39*
17	Control	7.51 ± 0.09	2.65 ± 0.09	0.21 ± 0.03	36.35 ± 0.65	1.47±0.04	27.25±0.44	18.59 ± 0.83	3.78 ± 0.05
	SSP	7.22 ± 0.08*	2.99 ± 0.08*	0.24 ± 0.02	35.59 ± 0.41	1.74±0.04*	30.48±0.33*	17.53 ± 0.40	18.54 ± 0.11*
24	Control	7.39 ± 0.06	2.44 ± 0.06	0.22 ± 0.02	35.11 ± 0.32	1.56±0.06	24.35±0.59	15.64 ± 0.88	4.78 ± 0.00
	SSP	7.04 ± 0.08*	3.19 ± 0.09*	0.23 ± 0.02	35.08 ± 0.62	1.84±0.02*	29.17±0.42*	15.83 ± 0.39	19.19 ± 0.06*
31	Control	7.34 ± 0.04	2.30 ± 0.06	0.34 ± 0.02	31.95 ± 0.74	1.78±0.05	23.06±0.46	12.99 ± 0.62	5.18 ± 0.01
	SSP	7.00 ± 0.02*	3.21 ± 0.05*	0.25 ± 0.03*	36.53 ± 1.04*	1.92±0.04	27.34±0.50*	14.25 ± 0.35	20.07 ± 0.12*
38	Control	7.35 ± 0.02	2.23 ± 0.05	0.48 ± 0.02	28.05 ± 0.66	1.84±0.05	22.77±0.12	12.37 ± 0.34	5.16 ± 0.02
	SSP	6.98 ± 0.02*	2.95 ± 0.11*	0.37 ± 0.02*	31.44 ± 1.02*	2.16±0.10	26.53±0.19*	12.31 ± 0.48	21.25 ± 0.10*

* Indicates significance at the 0.05 probability level.

Table S2

The diversity of methanogenic and methanotroph communities as affected by different fertilization during the compost. Different letters among treatments indicate significant difference at $P < 0.05$ possibility level.

	Treatments	Ace	Chao1	Shannon
Methanogen	Control-Day 5	152.12 ± 5.62	148.25 ± 7.45	3.00 ± 0.35
	CaSSP-Day 5	133.13 ± 17.56	129.09 ± 16.31	3.17 ± 0.22
	Control-Day 31	176.63 ± 15.05	167.69 ± 13.87	3.33 ± 0.29
	CaSSP-Day 31	158.77 ± 10.15	154.95 ± 10.43	3.42 ± 0.09
Methanotrophic	Control-Day 5	67.34 ± 1.68	61.05 ± 0.57	2.32 ± 0.11
	CaSSP-Day 5	70.87 ± 18.04	60.44 ± 3.46	2.23 ± 0.15
	Control-Day 31	39.98 ± 0.37	37.40 ± 1.65	1.51 ± 0.14
	CaSSP-Day 31	21.10 ± 9.54*	19.73 ± 8.11*	0.92 ± 0.15*

* Indicates significance at the 0.05 probability level.

Table S3

Methanogenic and methanotrophic community structures for tests of manure properties using the standard Mantel test.

	Methanogen		Methanotrophic	
	r	*p*	*r*	*p*
NH_4^+-N	0.152	0.138	0.275	**0.040**
NO_3^--N	-0.085	0.699	-0.090	0.744
pH	0.045	0.329	0.342	**0.020**
TN	0.325	0.024	0.747	<0.001
TC	0.461	0.005	0.540	0.003
C/N	0.487	0.002	0.712	0.002
SO_4^{2-}	0.211	0.049	0.139	0.102
DOC	0.337	0.023	0.085	0.246
CH_4	0.483	0.006	0.316	0.017
mcrA	0.447	**0.001**	0.800	**0.001**
pmoA	-0.054	0.602	0.579	**0.001**
